# Epidemiology, microbiological characteristics, and antimicrobial resistance of device-associated healthcare-associated infections in a neonatal intensive care unit: a six-year surveillance study

**DOI:** 10.3389/fpubh.2026.1979471

**Published:** 2026-10-30

**Authors:** Haiqiao Zhang, Mingyan Zou, Xiaoming Guo, Jiayu Cai, Zidong Liang

**Affiliations:** Department of Hospital Infection Management, Dongguan Maternal and Child Health Care Hospital, Dongguan, China

**Keywords:** antimicrobial resistance, central line-associated bloodstream infection, device-associated infections, neonatal intensive care unit, ventilator-associated pneumonia

## Abstract

**Background:**

Device-associated healthcare-associated infections (DA-HAIs) remain an important concern in neonatal intensive care units (NICUs). This study describes six-year surveillance data on the epidemiology, device utilization, pathogen distribution, and antimicrobial resistance of DA-HAIs.

**Methods:**

We conducted a retrospective descriptive surveillance study from 2020 to 2025. Ventilator-associated pneumonia (VAP), central line-associated bloodstream infection (CLABSI), and catheter-associated urinary tract infection (CAUTI) were classified using a standardized CDC/NHSN surveillance framework. Annual infection rates per 1,000 device-days, exact 95% confidence intervals (CIs), device utilization ratios, and isolate-level microbiological data were summarized.

**Results:**

Twenty-four CLABSI episodes occurred during 17,033 central-line days (1.41 per 1,000 line-days; 95% CI, 0.90–2.10), and 46 VAP episodes occurred during 21,628 ventilator-days (2.13 per 1,000 ventilator-days; 95% CI, 1.56–2.84). No CAUTI episodes occurred during 2,294 urinary catheter-days (95% CI, 0.00–1.61 per 1,000 catheter-days). Coagulase-negative staphylococci predominated among CLABSI isolates. Gram-negative organisms accounted for 25 of 42 recorded VAP isolates, while *Staphylococcus haemolyticus* was the single most common VAP isolate. MDR classifications varied by organism.

**Conclusion:**

These surveillance data characterize local DA-HAI patterns and support continued attention to device stewardship, microbiological surveillance, and antimicrobial stewardship.

## 1 Introduction

Neonates, particularly preterm and low-birth-weight infants, are highly susceptible to infection because of immature immune defenses and underdeveloped skin and mucosal barriers (1). In the NICU, mechanical ventilation, central venous catheters, enteral nutrition tubes, and other life-supporting equipment improve survival among critically ill newborns but are also relevant to healthcare-associated infection surveillance.

Device-associated infections constitute a major component of healthcare-associated infections in NICUs, including VAP and CLABSI (2, 3). Neonates often require these devices, which may provide opportunities for microbial colonization and biofilm formation and complicate treatment (3). Previous studies have linked DA-HAIs with prolonged hospitalization, antimicrobial exposure, and adverse outcomes.

Therefore, the present study aims to systematically describe the epidemiological characteristics of device-associated infections among neonates admitted to the NICU, including their incidence, demographic, and pathogen profiles. The findings are expected to provide evidence to support targeted infection prevention and control strategies and to lay the foundation for future mechanistic and interventional studies.

## 2 Materials and methods

### 2.1 Definitions of DA-HAIs

DA-HAIs were classified using the CDC/NHSN Patient Safety Component Manual; one standardized 2025 surveillance framework was applied retrospectively across 2020–2025 (4). A CLABSI required a laboratory-confirmed primary bloodstream infection, exclusion of a secondary bloodstream infection, and an eligible central line in place for more than 2 consecutive calendar days on the date of event or the preceding day. For common commensals, neonatal LCBI criteria required compatible neonatal signs or symptoms and two appropriately collected blood specimens yielding the same commensal within the infection window. VAP required the applicable CDC/NHSN PNEU/VAP imaging and clinical or laboratory criteria in a mechanically ventilated neonate; an endotracheal aspirate culture alone did not establish VAP. For all device-associated infections, the date-of-event and day-after-removal attribution rules were applied. The present study was a retrospective review of institutional surveillance records.

### 2.2 Microbiological testing

For symptomatic neonates, blood, lower-respiratory-tract, or urine specimens were collected in routine clinical care. Organisms were identified by standard laboratory methods. Antimicrobial susceptibility testing was performed by disk diffusion and interpreted according to the contemporaneous Clinical and Laboratory Standards Institute (CLSI) criteria. For bacterial isolates, MDR was defined as nonsusceptibility to at least one agent in three or more antimicrobial categories; intrinsic resistance was excluded. *Candida albicans* was reported separately and was not assigned a bacterial MDR classification (5, 6).

### 2.3 Statistical analysis

Counts and percentages summarized categorical data; medians and interquartile ranges summarized non-normally distributed continuous data. Annual DA-HAI rates were calculated as episodes per 1,000 device-days; exact Poisson 95% CIs were calculated using the Garwood method. Device utilization ratios were calculated as device-days divided by patient-days.

Analyses were performed using SPSS version 25.0 (IBM Corp., Armonk, NY, USA) and R version 4.5.3.

## 3 Results

### 3.1 Device-associated infections

Between 2020 and 2025, 24 CLABSI episodes occurred during 17,033 central-line days, yielding an overall rate of 1.41 per 1,000 line-days (95% CI, 0.90–2.10). Central-line utilization ratios ranged from 0.072 to 0.113 annually. Forty-six VAP episodes occurred during 21,628 ventilator-days, yielding an overall rate of 2.13 per 1,000 ventilator-days (95% CI, 1.56–2.84); annual ventilator utilization ratios ranged from 0.075 to 0.159. No CAUTI episode was observed during 2,294 urinary catheter-days; the corresponding rate was 0.00 per 1,000 catheter-days (95% CI, 0.00–1.61). All annual rates, CIs, and utilization ratios are shown in Table 1.

**Table 1:** Annual device-associated infection rates, exact 95% confidence intervals, and device utilization ratios in a neonatal intensive care unit, 2020–2025.

Measure	2020	2021	2022	2023	2024	2025	Total
CLABSI							
CLABSI infections	1	2	1	3	11	6	24
Central-line days	3,230	2,041	2,504	2,287	3,688	3,283	17,033
Patient-days	28,663	28,471	32,230	31,876	38,746	37,934	197,920
CLABSI rate per 1,000 line-days (95% CI)	0.31 (0.01–1.72)	0.98 (0.12–3.54)	0.40 (0.01–2.23)	1.31 (0.27–3.83)	2.98 (1.49–5.34)	1.83 (0.67–3.98)	1.41 (0.90–2.10)
Central-line utilization ratio	0.113	0.072	0.078	0.072	0.095	0.087	0.086
VAP							
VAP infections	10	9	5	8	8	6	46
Ventilator-days	3,691	4,242	5,121	2,583	2,896	3,095	21,628
Patient-days	28,663	28,471	32,230	31,876	38,746	37,934	197,920
VAP rate per 1,000 ventilator-days (95% CI)	2.71 (1.30–4.98)	2.12 (0.97–4.03)	0.98 (0.32–2.28)	3.10 (1.34–6.10)	2.76 (1.19–5.44)	1.94 (0.71–4.22)	2.13 (1.56–2.84)
Ventilator utilization ratio	0.129	0.149	0.159	0.081	0.075	0.082	0.109
CAUTI							
CAUTI infections	0	0	0	0	0	0	0
Urinary catheter-days	137	268	607	418	523	341	2,294
Patient-days	28,663	28,471	32,230	31,876	38,746	37,934	197,920
CAUTI rate per 1,000 catheter-days (95% CI)	0.00 (0.00–26.93)	0.00 (0.00–13.76)	0.00 (0.00–6.08)	0.00 (0.00–8.83)	0.00 (0.00–7.05)	0.00 (0.00–10.82)	0.00 (0.00–1.61)
Urinary-catheter utilization ratio	0.005	0.009	0.019	0.013	0.013	0.009	0.012

Rates are episodes per 1,000 device-days. Exact 95% CIs were calculated using the Garwood method. Device utilization ratio = device-days/patient-days.

### 3.2 Weight-stratified analysis of CLABSI and VAP

For CLABSI, the highest infection rate was observed in the >2,500 g group (1.989 per 1,000 catheter-days), followed by the 1,001–1,500 g group (1.356 per 1,000 catheter-days). The lowest CLABSI rate occurred in the ≤1,000 g group (0.730 per 1,000 catheter-days), despite this group having a relatively long cumulative catheter-duration (2,738 days). For VAP, the pattern differed. The ≤1,000 g group had the highest VAP rate (4.063 per 1,000 ventilator-days), which was more than double that of the >2,500 g group (2.051 per 1,000 ventilator-days). The lowest VAP rate was found in the 1,501–2,500 g group (1.340 per 1,000 ventilator-days). Overall, the ≤1,000 g group exhibited a disproportionately high VAP burden, while the >2,500 g group showed the highest CLABSI rate (Table 2).

**Table 2:** Weight-stratified number of episodes, device-days, and device-associated infection rates in a neonatal intensive care unit, 2020–2025.

Weight	CLABSI	Central-line days	CLABSI rate per 1,000 line-days	VAP	Ventilator days	VAP rate per 1,000 ventilator-days
≤1,000 g	2	2,738	0.730	14	3,446	4.063
1,001–1,500 g	6	4,426	1.356	5	3,210	1.558
1,501–2,500 g	4	3,837	1.042	7	5,223	1.340
>2,500 g	12	6,032	1.989	20	9,749	2.051

### 3.3 Demographic and clinical features of device-associated infections

Gender distribution differed between the two infection types. Among CLABSI cases, the gender distribution was equal (50% female, 50% male). In contrast, VAP cases showed a male predominance (71.74%, vs. 28.26% female).

Birth-weight strata showed different descriptive distributions. Most CLABSI episodes occurred in the >2,500-g group (12/24, 50.0%), followed by the 1,001–1,500-g group (6/24, 25.0%). In VAP, the >2,500-g group had the largest proportion of cases (20/46, 43.48%), whereas the ≤1,000-g group accounted for 14/46 cases (30.43%) and had the highest rate per 1,000 ventilator-days (4.06). Gestational-age distributions and median lengths of stay and ventilator days are presented in Table 3. These case distributions are descriptive and should not be interpreted as relative risks.

**Table 3:** Characteristics of CLABSI and VAP cases.

Characteristic	CLABSI *n* = 24, *n* (%) or median (IQR)	VAP *n* = 46, *n* (%) or median (IQR)
Gender		
Female	12 (50.00%)	13 (28.26%)
Male	12 (50.00%)	33 (71.74%)
Stratified by birth weight (g)		
≤1,000 g	2 (8.33%)	14 (30.43%)
1,001–1,500 g	6 (25.00%)	5 (10.87%)
1,501–2,500 g	4 (16.67%)	7 (15.22%)
>2,500 g	12 (50.00%)	20/46 (43.48%)
Gestational age (wk)		
≤ 27	3 (12.50%)	24/46 (52.17%)
28–31	12 (50.00%)	16/46 (34.78%)
32–36	8 (33.33%)	3/46 (6.52%)
≥ 37	1 (4.17%)	3/46 (6.52%)
Length of hospital stay (d)		
≤1,000 g	89.00 (84.00, 94.00)	94.50 (61.25, 114.75)
1,001–1,500 g	21.00 (17.75, 56.50)	65.00 (48.00, 72.00)
1,501–2,500 g	39.00 (23.25, 51.25)	59.00 (44.00, 69.00)
>2,500 g	55.00 (39.00, 71.00)	70.00 (65.50, 85.25)
Ventilator days (d)		
≤1,000 g	33.50 (28.25, 38.75)	43.00 (31.00, 53.75)
1,001–1,500 g	16.50 (9.75, 27.75)	37.00 (15.00, 41.00)
1,501–2,500 g	10.50 (8.75, 14.25)	20.00 (9.50, 30.50)
>2,500 g	16.50 (12.00, 31.00)	35.00 (22.75, 52.50)

Values are *n* (%) or median (IQR), as indicated. CLABSI, central line-associated bloodstream infection; VAP, ventilator-associated pneumonia.

### 3.4 Microbiology and antimicrobial resistance patterns in CLABSI and VAP

Microbiological data are reported at the isolate level (Table 4). Twenty-five isolates were recovered from 24 CLABSI episodes, indicating at least one polymicrobial episode. Forty-two isolates were recorded from 46 VAP episodes; pathogen frequencies therefore do not represent all VAP episodes. CoNS accounted for most CLABSI isolates, with *Staphylococcus epidermidis* and *S. hominis* each identified in five isolates. In VAP, Gram-negative organisms accounted for 25 of 42 recorded isolates (59.5%); however, *S. haemolyticus* (10/42, 23.8%) was the single most frequently recorded isolate. *E. coli* and *K. pneumoniae* each accounted for six VAP isolates, of which five were classified as MDR. No MDR isolate was identified among the *A. baumannii* or *P. aeruginosa* isolates. *Candida albicans* was reported separately without MDR classification.

**Table 4:** Distribution of recorded isolates and multidrug-resistance classifications for CLABSI and VAP.

Organism	CLABSI isolates (*n* = 25)		VAP isolates (*n* = 42)	
	No. of isolates	MDR *n*/*N* (%)	No. of isolates	MDR *n*/*N* (%)
Gram-positive pathogens				
*Staphylococcus epidermidis*	5	4/5 (80.0%)	0	—
*Staphylococcus hominis*	5	5/5 (100.0%)	0	—
*Staphylococcus haemolyticus*	3	3/3 (100.0%)	10	9/10 (90.0%)
*Staphylococcus capitis*	2	2/2 (100.0%)	0	—
*Enterococcus faecalis*	1	0/1 (0.0%)	0	—
*Enterococcus faecium*	1	0/1 (0.0%)	0	—
*Staphylococcus aureus* subsp. aureus	0	—	5	0/5 (0.0%)
*Staphylococcus lugdunensis*	0	—	1	0/1 (0.0%)
Gram-negative pathogens				
*Escherichia coli*	2	2/2 (100.0%)	6	5/6 (83.3%)
*Acinetobacter junii*	2	0/2 (0.0%)	1	0/1 (0.0%)
*Pseudomonas putida*	1	1/1 (100.0%)	0	—
*Klebsiella pneumoniae*	1	0/1 (0.0%)	6	5/6 (83.3%)
*Enterobacter cloacae*	1	0/1 (0.0%)	3	0/3 (0.0%)
*Acinetobacter nosocomialis*	1	1/1 (100.0%)	0	—
*Acinetobacter baumannii*	0	—	4	0/4 (0.0%)
*Pseudomonas aeruginosa*	0	—	4	0/4 (0.0%)
*Stenotrophomonas maltophilia*	0	—	1	0/1 (0.0%)
Fungi				
*Candida albicans*	—	—	1	Not assessed

CLABSI, central line-associated bloodstream infection; VAP, ventilator-associated pneumonia; MDR, multidrug resistance; CoNS, coagulase-negative staphylococci. MDR was classified for bacterial isolates only, as defined in the Methods. Percentages are MDR isolates / isolates of the same species. Data are isolate-level: 25 isolates were recovered from 24 CLABSI episodes and 42 isolates were recorded from 46 VAP episodes; at least one CLABSI episode was polymicrobial.

## 4 Discussion

Over 6 years, VAP and CLABSI were the recorded DA-HAIs, while no CAUTI episode was observed. The overall VAP and CLABSI rates were 2.13 (95% CI, 1.56–2.84) and 1.41 (95% CI, 0.90–2.10) per 1,000 device-days, respectively. These results provide a local surveillance benchmark and are broadly comparable with published neonatal device-associated infection surveillance data (2, 7).

Annual rates varied, but the event counts were small and their 95% CIs were wide. The descriptive changes across years should not be interpreted as evidence that any prevention measure caused a decline, because intervention timing, implementation, adherence, and a formal interrupted time-series or similar evaluation were not available.

Preterm and extremely low-birth-weight neonates have well-recognized vulnerability to healthcare-associated infections because of immature host defenses and the need for intensive support (1, 7). In the present descriptive data, the ≤1,000-g group had the highest VAP rate; this does not establish a causal association.

An initial risk-factor analysis was considered but was not retained after the data audit. The marked baseline imbalances, particularly in VAP, and the absence of index-date-specific device exposure would have made risk-factor estimates vulnerable to confounding, opportunity time, and reverse causation. Device duration, delivery mode, and other baseline variables should therefore not be interpreted as independent predictors in this study. Future studies should use risk-set sampling or a time-to-event design beginning at device insertion, with pre-specified covariates and appropriate accounting for device removal, discharge, and death.

CoNS were the most frequently recorded CLABSI isolates. Given the prominence of CoNS, rigorous application of common-commensal criteria and exclusion of secondary BSI are important in surveillance interpretation (4).

For VAP, Gram-negative organisms represented the largest class of recorded isolates, whereas *S. haemolyticus* was the most common individual organism. The isolate-level counts should be interpreted cautiously because 42 isolates were recorded for 46 VAP episodes, and recovery from a respiratory specimen did not by itself establish VAP.

MDR classifications were frequent among several CoNS and among the *E. coli* and *K. pneumoniae* isolates. The analysis is based on small organism-specific denominators and is reported as n/N throughout Table 4. The data do not permit claims of full susceptibility for organisms with no MDR classification. MDR S. haemolyticus has also been reported in neonatal intensive care settings (8). Antimicrobial-resistant Enterobacterales are an important concern in NICU surveillance, including ESBL-producing *E. coli* and *K. pneumoniae* (9).

These isolate-level findings underscore the value of local antimicrobial stewardship, microbiological surveillance, and infection-prevention practice. However, they do not establish a mechanism of resistance acquisition or transmission.

Uncommon Acinetobacter species, including *A. junii* and *A. nosocomialis*, were recorded. Non-baumannii Acinetobacter species have been reported in neonatal infections (10), and A. junii bacteremia has been described in a hospital case series (11). The present surveillance data cannot determine their virulence, resistance mechanism, or transmission route.

Acinetobacter species can persist in healthcare environments and form biofilms, but no environmental or genomic investigation was performed in this study (10, 12). Enhanced environmental hygiene, standard device care, and targeted microbiological review are reasonable infection-prevention priorities.

Previous neonatal studies have examined the clinical utility of routine central-catheter tip cultures (13) and risk factors associated with VAP (14). These reports provide clinical context; independent risk factors were not evaluated in the present study.

No CAUTI episode was observed over 2,294 urinary catheter-days. The corresponding 95% CI for the rate was 0.00–1.61 per 1,000 catheter-days. This observation should be interpreted cautiously because zero observed events do not establish the absence of CAUTI.

Several limitations should be acknowledged. First, this was a retrospective single-center study, which may limit the generalizability of our findings. Future multicenter prospective studies with standardized surveillance protocols are needed to validate these results across different NICU settings. Second, annual event counts were small, limiting the interpretation of temporal trends. Pooled multi-year and multi-center data would provide more robust estimates of annual infection rates and trends. Third, because the number of device-associated infection events was limited (24 CLABSI and 46 VAP), formal risk-factor modeling was not performed; therefore, independent predictors could not be reliably identified, and residual confounding cannot be excluded. Future studies with larger sample sizes, multicenter collaboration, and pre-specified time-to-event or risk-set analyses are needed to evaluate independent predictors for VAP and CLABSI. Fourth, four VAP episodes had no recorded isolate; these episodes were not classified as culture-negative because negative culture results were not documented in the surveillance dataset. They were diagnosed as VAP based on clinical and radiographic criteria after excluding other infectious etiologies. Although this is consistent with accepted VAP surveillance definitions, the absence of a causative isolate may have limited the completeness of the pathogen profile. Future studies should incorporate standardized lower respiratory tract sampling, quantitative cultures, and molecular diagnostic methods to improve pathogen detection. Finally, molecular mechanisms of antimicrobial resistance and clinical outcomes, including attributable mortality and long-term neurodevelopmental outcomes, were not evaluated. Future research should integrate genomic and clinical outcome data to better understand resistance mechanisms and long-term prognosis. These findings should be interpreted as descriptive and hypothesis-generating.

In conclusion, this six-year NICU surveillance study describes event rates, device-utilization patterns, and differing isolate distributions for CLABSI and VAP. The results should be interpreted as descriptive surveillance data rather than evidence of causal risk factors. Ongoing standardized surveillance, device stewardship, microbiological review, and antimicrobial stewardship remain important.

## Data Availability

The original contributions presented in the study are included in the article/supplementary material, further inquiries can be directed to the corresponding author.
